# Effect of dietary approaches to stop hypertension and curcumin co-administration on glycemic parameters in polycystic ovary syndrome: An RCT

**DOI:** 10.18502/ijrm.v22i9.17473

**Published:** 2024-11-14

**Authors:** Tayebeh Zohrabi, Azadeh Nadjarzadeh, Sara Jambarsang, Mohammad Hasan Sheikhha, Abbas Aflatoonian, Hassan Mozaffari-Khosravi

**Affiliations:** ^1^Department of Nutrition, School of Public Health, Shahid Sadoughi University of Medical Sciences, Yazd, Iran.; ^2^Research Center for Food Hygiene and Safety, School of Public Health, Shahid Sadoughi University of Medical Sciences, Yazd, Iran.; ^3^Center for Healthcare Data Modeling, Department of Biostatistics and Epidemiology, School of Public Health, Shahid Sadoughi University of Medical Sciences, Yazd, Iran.; ^4^Department of Genetics, Faculty of Medicine, Shahid Sadoughi University of Medical Sciences, Yazd, Iran.; ^5^Abortion Research Center, Yazd Reproductive Sciences Institute, Shahid Sadoughi University of Medical Sciences, Yazd, Iran.; ^6^Research and Clinical Center for Infertility, Yazd Reproductive Sciences Institute, Shahid Sadoughi University of Medical Sciences, Yazd, Iran.

**Keywords:** Polycystic ovary syndrome, Dietary approaches to stop hypertension, Curcumin, Insulin resistance, Glycemic control.

## Abstract

**Background:**

olycystic ovary syndrome (PCOS) is an endocrine disorder that can lead to major reproductive and metabolic complications. Management of glycemic parameters is an important step to improve the symptoms of PCOS.

**Objective:**

This study aims to evaluate the effects of the dietary approaches to stop hypertension (DASH) diet and curcumin (Cur) co-administration on glycemic parameters in normal weight and overweight/obese women with PCOS undergoing in vitro fertilization.

**Materials and Methods:**

In this double-blind randomized clinical study, 104 infertile women with PCOS were divided into 4 intervention groups considering treatment conditions and body mass index. They received 500 mg twice daily of Cur or placebo (Pls) along with DASH or a standard diet (St) based on macronutrient composition (52% carbohydrate, 18% protein, and 30% fat) for 12 wk, (DASH + Cur, DASH + Pls, St + Cur, St + Pls). The effect of these interventions on fasting blood sugar and insulin levels, insulin resistance (IR), and insulin sensitivity were determined.

**Results:**

Participants adhered to the intervention protocol ( > 80%). Insulin levels in the supplement intervention (Cur + diet) decreased significantly in the crude model. They remained significant even after adjusting for confounding variables in adjusted models (body mass index classification, energy difference, age, and physical activity levels at the baseline) ( β = -45.3, 95% CI [-73.23, -17.46], p = 0.002). Homeostasis model assessment of IR decreased significantly in the supplement intervention even after controlling for confounding factors in adjusted models. Changes in fasting blood sugar and insulin sensitivity were not significant in either the crude or adjusted models.

**Conclusion:**

The study results show that adding Cur to the diet can positively reduce insulin levels, improve IR, and lead to faster recovery of hyperinsulinemia. Cur supplementation with a healthy diet has synergistic beneficial effects on glycemic parameters. Larger clinical trials with longer durations are needed to confirm these results.

## 1. Introduction

Polycystic ovary syndrome (PCOS) is an endocrine disorder that can lead to major reproductive and metabolic consequences (1). This complex disorder, which can be recognized by menstrual disorder, polycystic ovarian morphology, hirsutism, acne, and infertility, significantly reduces the patient's quality of life (2).

About 50–70% of people with PCOS are at high risk for insulin resistance (IR) (3). Insulin plays an important role in regulating glucose homeostasis by stimulating the uptake of glucose into skeletal muscles and fat tissues, and inhibiting glucose production in the liver (4). IR is a condition in which the body's cells do not respond properly to insulin (5). In these conditions, the pancreas produces more insulin to overcome the resistance, resulting in hyperinsulinemia, which can be the primary mediator of metabolic syndrome, leading to type 2 diabetes and cardiovascular diseases (6).

Various methods have been proposed to control IR (2, 7). Lifestyle intervention is the first line of IR prevention in prediabetic and diabetic conditions (8). Dietary intervention can include a wide variety of caloric restriction patterns or the use of a low glycemic index diet (7). It has been suggested that dietary approaches to stop hypertension (DASH) is an effective diet with a low glycemic index and high antioxidant content that can have beneficial effects in reducing IR (9). The DASH diet is rich in whole grains, vegetables, fruits, low-fat dairy products, poultry, fish, and nuts while limiting the intake of foods high in saturated fat, cholesterol, sodium, refined grains, red and processed meat (10). Consuming the DASH dietary pattern in overweight and obese women with PCOS had favorable effects on IR, abdominal fat accumulation, and high-sensitivity C-reactive protein serum level (11).

A variety of other interventions and many potential compounds with different therapeutic activities have been investigated in recent years. In particular, curcumin's therapeutic properties and antioxidant effects as natural plant polyphenols have received more attention (12). Curcumin (diferroylmethane) is a lipophilic yellow pigment extracted from the rhizomes of turmeric (*Curcuma longa* L.) (13). Findings show that curcumin supplementation can affect glucose homeostasis, improve lipid metabolism and antioxidant capacity, and reduce oxygen radical species in PCOS patients (14).

Investigating the combined effects of using healthy dietary patterns and natural bioactive compounds will help researchers, nutritionists, and policymakers prescribe optimal modifications to improve quality of life and reduce PCOS-related complications. Therefore, the objective of the present clinical trial was to investigate the effect of the DASH diet along with curcumin supplementation on glycemic parameters in normal weight and overweight/obese PCOS patients undergoing in vitro fertilization (IVF) treatment. The preprint version of the present article has been uploaded to the Research Square (https://www.researchsquare.com/article/rs-3610838/v1, https://doi.org/10.21203/rs.3.rs-3610838/v1).

## 2. Materials and Methods

This double-blind, randomized clinical study was conducted on 104 infertile women with PCOS at the Gynecology Clinic of Research and Clinical Center for Infertility affiliated to Yazd Reproductive Sciences Institute of Shahid Sadoughi University of Medical Sciences, Yazd, Iran from August 2022 to April 2023.

Infertile women with PCOS, according to Rotterdam's criteria (15), based on the inclusion criteria (without a previous IVF history, between 18 and 45 yr, and with body mass index [BMI] between 18.5 and 25 kg and BMI ≥ 25 kg/m²), and exclusion criteria were included in the study.

This study was a 12-wk, randomized, placebo-controlled clinical trial with a factorial design. Participants were assigned unique codes to conceal the allocation and avoid “selection bias”. They were arranged in 4 blocks of equal size using the stratified permuted block randomization method by the “blockrand” package in R software, version 4.0.2.

Treatment conditions and BMI (18.5–25 and ≥ 25) were included in the random allocation list. Supplemental and placebo capsules were serially placed in numbered and sealed envelopes by a person unrelated to the study. These capsules were packaged in similar bottles with the same appearance (in terms of color and shape).

The bottles had different marks (A and B). Participants and researchers were not aware of the contents of the bottles. Randomization and allocation were kept secret from participants and researchers until the end of the study and data analysis. Participants were selected using the available sampling method and 2 × 2 factorial design, and randomly divided into 4 parallel treatment groups (n = 26/each group): 1) DASH diet + curcumin supplement, 2) standard diet + curcumin supplement, 3) DASH diet + placebo, and 4) standard diet + placebo.

Details of the method have been published previously. This manuscript is part of a larger clinical trial that includes part of the data. Other parameters (genetic, androgenic, nutritional, etc.) were evaluated according to the protocol. But, due to the high volume of data and to prevent the increase in the number of tables and words, they will be discussed in other articles. It should also be noted that according to the sample size formula that is fully explained in the study protocol (considering an effect size = 1, α = 0.05, power of 80%, and dropout rate of 20%) (16), the optimal sample size was 24 people in each group. However, after the start of the study, due to the distance problems of many participants and the concern of follow-up, the research team decided to add 2 people to the sample size of each group. The final volume was considered to be 104 people. 


$$n=\frac{{{(Z}_{1-\frac{\alpha }{2}}+Z_{1-\beta })}^{2}\left(S_{1}^{2}+S_{2}^{2}\right)}{{\left(\mu_{1}-\mu_{2}\right)}^{2}}$$

### Intervention

Participants received 500 mg of placebo (roasted rice powder) or curcumin capsules (BCM95/Curcugreen) twice daily, once after breakfast and once after dinner with water. Participants received 60 capsules each month. Each 500 mg capsule contains 475 mg of curcuminoids along with natural turmeric essential oil, which improves the bioavailability and bioactivity of oral curcuminoids. All capsules were manufactured by M/s Arjuna Natural Pvt Ltd., India. Compliance was estimated by collecting bottles at the end of the month and counting the remaining capsules. In addition, all volunteers followed 2 healthy eating patterns (DASH or standard diet). These individual diets were designed in the form of diets containing 18% protein, 52% carbohydrates, and 30% total fat. Daily caloric needs were estimated by calculating physical activity levels and resting energy expenditure. Calculated energy was reduced by 300 and 500 kcal, respectively, if a person was overweight (BMI 25.0–30.0 kg/m²) or obese ( > 30.0 kg/m²). A similar amount of sodium (about one teaspoon of salt) was prescribed for each diet. Macronutrients and energy intake were obtained using 3-day dietary records (including 2 nonconsecutive weekdays and one weekend) and analyzed by Nutritionist IV software. Adherence to the DASH dietary pattern was evaluated based on Dixon's DASH dietary index (17). Dixon's recommendations for cut-off values based on 1600 kcal per day for women in 9 components are as follows:

Whole grains ≥ 4 servings per day (67%* 6 servings of grains), ≥ 4 servings per day of fruits, ≥ 2 servings per day of dairy products, ≥ 3 servings per day of vegetables, ≥ 3 servings per day of seeds, nuts and legumes, < 6 ounces per day of meat and meat equivalents, average daily intake of saturated fat < 5% of total energy intake, added sugar intake ≤ 3% of total energy intake, alcohol intake < one drink per day. The minimum score is 0, and the maximum score is 9 points. The sum of 9 components was considered as the total score of people's adherence to the DASH diet.

Physical activity was assessed with the metabolic equivalents questionnaire at the beginning and end of the study, although all volunteers were advised not to change their physical activity during the study.

### Anthropometric and biochemical measurements

Biochemical and anthropometric assessments were done at the beginning and after 12 wk of intervention. After a 2-wk run period and considering 12 hr overnight fasting, 5 ml of blood sample was collected from each participant in the morning. The serum was removed from centrifuged samples and immediately stored at -70 C. The serum levels of fasting insulin were assessed using an enzyme-linked immunosorbent assay kit (Monobind, California, United States). Fasting blood sugar (FBS) was measured using an autoanalyzer (AVIDA 1800 chemistry system; Siemens, United Kingdom, with Pars Azmoon Kits). Homeostasis model assessment of IR (HOMA-IR) and quantitative insulin sensitivity check index (QUICKI) were also calculated.

Body composition and weight were determined using a segmental body composition analyzer (Tanita BC-418, Tokyo, Japan). Height, hip, and waist circumference measurements were done according to the standard protocol. BMI was calculated by dividing weight in kilograms by the square of height in meters (BMI = kg/m²).

### Ethical Considerations

This research was approved by the Medical Ethics Committee of Shahid Sadoughi University of Medical Sciences, Yazd, Iran (Code: IR.SSU.SPH.REC.1399.145). The protocol of this trial with the registration ID: IRCT20200915048731N1 and URL: https://www.irct.ir/trial/50970 was registered on the Iran Clinical Trials website on 09/29/2020. It has been updated on 02 May 2024. In this research, all methods and experiments were performed in accordance with the Declaration of Helsinki, and the relevant guidelines and regulations. This study was also conducted in accordance with the consolidated standards for reporting trials (CONSORT). Written informed consent was signed by all participants before the start of the study.

### Statistical Analysis

Before statistical analysis, all data were checked for completeness and accuracy. The Kolmogorov-Smirnov test evaluated the normal distribution of the data. The Chi-square test was used to compare the frequency between the studied groups. The changes before and after the intervention in each group were performed using the paired *t* test/Wilcoxon test. Comparison between groups was evaluated through one-way analysis of variance (ANOVA)/Kruskal Wallis. Analysis of covariance (ANCOVA) was performed to compare the mean of quantitative data between groups after adjustment for potential confounders. The values of quantitative variables were expressed as mean ± standard deviation/MD, IQR and qualitative variables were expressed as numbers and percentages. A p ≤ 0.05 was defined as statistically significant. All statistical analyses were performed using SPSS (SPSS, Inc, Chicago, IL, USA), version 25.0.

## 3. Results

**Figure 1:**
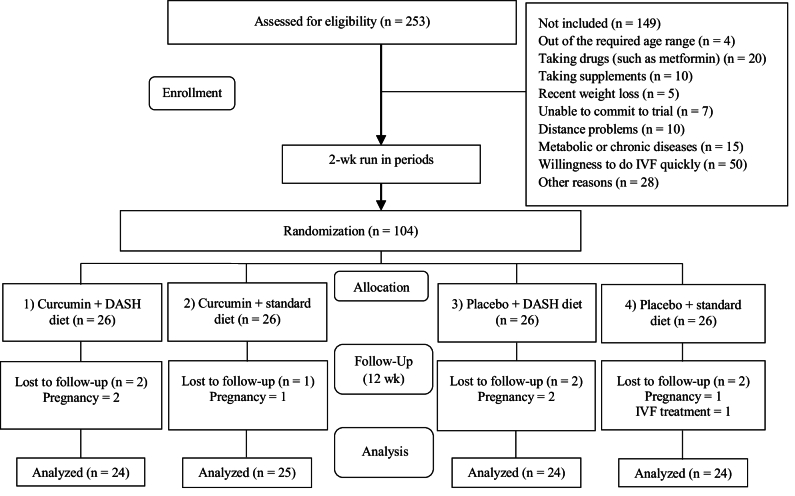
CONSORT flow diagram of eligibility, screening, and follow-up of participant.

253 participants were evaluated for eligibility. Based on the inclusion and exclusion criteria, 104 volunteers were included in the study. 149 participants were not included due to reasons such as being out of the required age range (n = 4), taking some drugs (n = 20) or supplements (n = 10), having recent weight loss (n = 5), unable to commit to trial (n = 7), distance problems (n = 10), metabolic or chronic diseases (n = 15), desire to do IVF quickly (n = 50), other reasons (n = 28). 7 participants were excluded from the study due to loss of follow-up (6 were excluded due to pregnancy and 1 due to IVF). Ultimately, a total of 97 participants completed the trial (Figure 1).

Adherence to the DASH diet based on the Dixon index in the DASH diet groups was, on average, between 7.8 and 8 (86.6–88.8%). All participants consumed the capsules completely, except one who did not consume 2 capsules due to a delay in returning at the appointed time. No complications were reported due to the use of capsules. Overall, no serious adverse events were reported during the study.

No significant difference was observed between the groups in terms of basic parameters for age examination, disease duration, education level, and height. Descriptive statistics of baseline parameters, physical activity, and daily dietary intake in intervention groups based on BMI classification are presented in table I. No significant difference was observed between the groups in terms of macronutrient composition including carbohydrates, protein, and fat with proportions of 52, 18, and 30%. Table II shows the comparison of glycemic parameters between and within groups. These parameters decreased significantly in each group compared to baseline (p < 0.05). The comparison of the average serum levels of glycemic parameters between the studied groups before the intervention did not show a statistically significant difference (p ≥ 0.05). However, a significant difference was observed regarding serum insulin and HOMA-IR after the intervention.

The effects of diet and supplement interventions on glycemic parameters are shown in (Table III). Using an unadjusted model, insulin levels were significantly reduced in the supplement intervention (p = 0.006). After controlling for “energy difference” and “BMI classification” in the first model (model I) (p = 0.001), they were significant even with further adjustments for age in model II (p = 0.002) and for baseline levels of physical activity in model III (p = 0.002). HOMA-IR decreased significantly in the supplement intervention (p = 0.004). The reduction of IR in the supplement intervention was significant even with further adjustments in model I (p = 0.001), model II (p = 0.002), and model III (p = 0.001). However, no significant difference was observed in glycemic parameters in the diet intervention in crude or adjusted models (p ≥ 0.05). Changes in FBS and QUICKI index were not significant in either the crude or adjusted models in the supplement intervention (p ≥ 0.05).

**Table 1:** Descriptive statistics of baseline parameters, physical activity, and daily dietary intake in intervention groups based on BMI category


**Variables**		**BMI 18.5–25** **kg/m** ^2^ ** (n = 48)**				**BMI ≥ 25 kg/m** ^2^ ** (n = 49)**				
		**DASH diet and curcumin**	**DASH diet and placebo**	**Curcumin and standard diet**	**Placebo and standard diet**	**DASH diet and curcumin**	**DASH diet and placebo**	**Curcumin and standard diet**	**Placebo and standard diet**	**P-value^#^**
**Age (yr) † **		29 ± 4.19 (29.5, 6.75)	28.3 ± 5.5 (31, 9)	29.2 ± 5.3 (29.5, 8.5)	27.1 ± 4.9 (27, 7)	27.9 ± 5.3 (27, 9)	29.6 ± 3.4 (30, 6.25)	28.4 ± 4.2 (27, 6.5)	26 ± 5.7 (24.5, 9.75)	
**Height (cm) † **		162 ± 4.9 (162.5, 7.5)	160 ± 7 (160, 10.5)	160 ± 4.7 (160, 6.5)	160 ± 9.1 (161, 11.25)	159 ± 7.9 (161, 11.25)	158 ± 3.6 (158, 5.13)	162 ± 5.9 (165, 9.5)	161 ± 4.2 (162, 7.5)	-
**PCOS duration**		6.4 ± 5.5 (4.25, 7.75)	5.8 ± 5.1 (4.5, 7.25)	5 ± 3.8 (4, 7)	4.2 ± 4.2 (2.75, 4.25)	5.8 ± 5 (3, 8)	8.2 ± 6.8 (5.5, 13)	6.7 ± 4.3 (5, 6.5)	5.5 ± 3.5 (5, 4.75)	-
**Waist to hip (cm)**										
	**Before**	0.798 ± 0.055	0.80 ± 0.34	0.77 ± 0.054	0.794 ± 0.0478	0.80 ± 0.043	0.79 ± 0.064	0.82 ± 0.059	0.81 ± 0.06	0.032
	**After**	0.790 ± 0.051	0.78 ± 0.026	0.77 ± 0.050	0.790 ± 0.0476	0.784 ± 0.045	0.776 ± 0.068	0.795 ± 0.067	0.80 ± 0.05	< 0.001
	P-value*	0.066	0.135	0.615	0.065	0.004	0.003	< 0.001	0.013	-
**BMI (kg/m^2^)**										
	**Before**	22.41 ± 1.84	23.96 ± 1.7	23.72 ± 2.3	23.76 ± 1.52	31.51 ± 3.85	32.02 ± 4.19	31.53 ± 3.2	31.99 ± 4.3	< 0.001
	**After**	22.13 ± 1.95	23.76 ± 1.57	23.53 ± 2.2	23.25 ± 1.73	28.81 ± 2.98	28.92 ± 3.68	28.72 ± 2.9	29.26 ± 3.39	< 0.001
	P-value*	0.323	0.318	0.268	0.065	< 0.001	< 0.001	< 0.001	< 0.001	-
**Energy intake (Kcal)**										
	**Before**	2110.4 ± 420.3	2601.68 ± 692.6	2213.3 ± 322.8	2173.1 ± 449.2	2987.2 ± 733.7	2538.3 ± 470.5	2458.3 ± 461.8	2766.5 ± 485	0.001
	**After**	1707.3 ± 97.9	1780.3 ± 169.6	1677.1 ± 82.5	1695.7 ± 163.6	1618.1 ± 169	1527.7 ± 123.7	1628.6 ± 155	1598.6 ± 115	0.001
	P-value*	0.012	0.001	< 0.001	0.002	< 0.001	< 0.001	< 0.001	< 0.001	-
**Percent of energy from protein**										
	**Before**	12 ± 2.55	11.78 ± 1.9	11.44 ± 1.74	12.7 ± 2.3	11.9 ± 2.76	11.89 ± 1.61	12.07 ± 3.06	10.58 ± 1.97	0.565
	**After**	18.19 ± 0.27	18.04 ± 0.19	17.96 ± 0.24	17.89 ± 0.24	18.15 ± 0.25	18.06 ± 0.34	17.87 ± 0.45	17.93 ± 0.36	0.106
	P-value*	< 0.001	< 0.001	< 0.001	< 0.001	< 0.001	< 0.001	< 0.001	< 0.001	-
**Percent of energy from carbohydrate**										
	**Before**	53.4 ± 9.3	49.2 ± 4.38	54 ± 7	50.8 ± 7	51.67 ± 5.2	48.17 ± 5.08	48.54 ± 11.9	51.13 ± 7.63	0.457
	**After**	52.07 ± 0.28	52.05 ± 0.37	52.01 ± 0.45	51. 8 ± 0.45	52.03 ± 0.42	52.02 ± 0.35	51.97 ± 0.16	51.88 ± 0.29	0.613
	P-value*	0.629	0.048	0.350	0.67	0.812	0.024	0.32	0.741	-
**Percent of energy from fat**										
	**Before**	33.4 ± 9.86	38 ± 4.7	31.8 ± 8.1	35.4 ± 6.97	36.28 ± 6.42	36.62 ± 11.25	39.06 ± 11.74	37.92 ± 8.25	0.468
	**After**	29.92 ± 0.35	29.94 ± 0.12	30.01 ± 0.18	30.2 ± 0.37	29.85 ± 0.22	29.99 ± 0.14	30.01 ± 0.43	30.11 ± 0.47	0.194
	P-value*	0.245	< 0.001	0.457	0.027	0.005	0.066	0.016	0.008	-
**Adherence to the DASH dietary pattern (Dixon's index)**										
	**Before**	4.16 ± 0.937	3.75 ± 0.75	4.25 ± 0.866	3.9 ± 0.9	4.4 ± 0.99	3.9 ± 0.9	4.07 ± 0.95	4.16 ± 1	-
	**After**	8 ± 0.66	7.8 ± 0.83	5.1 ± 0.71	5.16 ± 0.71	7.8 ± 1.11	7.9 ± 1.16	5 ± 0.7	5.5 ± 0.67	-
**Physical activity (MET-h/wk)**										
	**Before**	30.97 ± 2.55	31.78 ± 1.25	30.4 ± 1.57	31.35 ± 1.56	31.32 ± 1.75	30.54 ± 2.11	30.96 ± 2.04	31.61 ± 2.77	0.666
	**After**	31.6 ± 0.92	31.47 ± 1.23	30.48 ± 1.36	31.54 ± 1.07	31.16 ± 1.62	31.06 ± 1.13	31.47 ± 1.15	31.11 ± 1.85	0.485
	P-value*	0.372	0.425	0.373	0.585	0.771	0.276	0.215	0.474	-
Educational level**										
	**Elementary**	3 (25)	4 (33.33)	1 (8.33)	1 (8.33)	4 (33.33)	1 (8.33)	3 (23)	6 (50)	0.731
	**Diploma**	3 (25)	5 (41.66)	5 (41.66)	6 (50)	4 (33.33)	7 (58.33)	7 (53.84)	4 (33.33)	
	**Academic** **education**	6 (50)	3 (25)	6 (50)	5 (41.66)	4 (33.33)	4 (33.33)	3 (23)	2 (16.66)	
** † ** Data were presented as Mean ± SD (MD, IQR). *Data were presented as Mean ± SD. Paired *t* test for the comparison variables within the group. ** ${\;}^{\#}$ ** Data were presented as Mean ± SD. One-way ANOVA for the comparison variables between groups. **Data were presented as n (%) by descriptive statistics: Chi-square. BMI: Body mass index, PCOS: Polycystic ovary syndrome, DASH: Dietary approaches to stop hypertension, MET: Metabolic equivalent task										

**Table 2:** The comparison of glycemic parameters between and within groups

**Variables**		**DASH diet and curcumin (n = 24)**	**DASH diet and placebo (n = 24)**	**Curcumin and standard diet (n = 25)**	**Placebo and standard diet (n = 24)**	**P-value^#^**
**FBS (mg/dl)**						
	**Before † **	98.2 ± 13.16 (93.5, 20.25)	104.1 ± 13.79 (102.5, 17.5)	98.38 ± 12.32 (98, 17.5)	100.96 ± 14.09 (100.5, 20.5)	0.38
	**After † **	82.93 ± 11.44 (84.7, 13)	87.91 ± 10.1 (90, 17.63)	85.24 ± 9.85 (86.5, 13.25)	88.45 ± 10.91 (90, 18)	0.248
	P-value*	< 0.001	< 0.001	< 0.001	< 0.001	-
**Insulin (μU/ml)**						
	**Before † **	221.67 ± 88.76 (247.15, 140.75)	238.64 ± 67.39 (238.5, 114.7)	236.89 ± 97.56 (286.9, 114)	245.35 ± 74.8 (262.9, 110.05)	0.791
	**After † **	39.2 ± 44.27 (27.9, 53.8)	70.06 ± 73.67 (33.01, 130.17)	45.61 ± 60.86 (19.4, 73.25)	104.19 ± 110.19 (46.6, 190.02)	0.016
	P-value*	< 0.001	< 0.001	< 0.001	< 0.001	-
**HOMA-IR**						
	**Before † **	55.01 ± 24.59 (61.5, 33.82)	61.74 ± 21.16 (55.84, 31.58)	58.03 ± 24.35 (69.22, 36.88)	61.44 ± 21.82 (63.9, 29.96)	0.713
	**After † **	6.9 ± 7.7 (5.09, 8.12)	13.46 ± 13.77 (6.69, 23.4)	8.96 ± 11.86 (3.11, 14.83)	20.19 ± 20.79 (10.57, 43.85)	0.009
	P-value*	< 0.001	< 0.001	< 0.001	< 0.001	-
**QUICKI index**						
	**Before † **	0.235 ± 0.021 (0.227, 0.01)	0.229 ± 0.008 (0.229, 0.01)	0.24 ± 0.05 (0.22, 0.02)	0.231 ± 0.017 (0.22, 0.01)	0.45
	**After † **	0.382 ± 0.239 (0.29, 0.08)	0.341 ± 0.167 (0.28, 0.14)	0.46 ± 0.32 (0.31, 0.23)	0.388 ± 0.299 (0.27, 0.1)	0.43
	P-value*	0.006	0.003	0.001	0.017	-
Data were presented as Mean ± SD. *Paired sample *t* test and Wilcoxon test for the comparison variables within the group. ${\;}^{\#}$ One-way ANOVA and Kruskal Wallis test for the comparison variables between groups. † Data presented as Mean ± SD (MD, IQR). DASH: Dietary approaches to stop hypertension, FBS: Fasting blood sugar, HOMA-IR: Homeostasis model assessment of insulin resistance, QUICKI: Quantitative insulin sensitivity check index						

**Table 3:** The effects of diet and supplement interventions on glycemic parameters

**Variables**		**crude β (CI)**	**P-value**	**Model I β (CI)**	**P-value**	**Model II β (CI)**	**P-value**	**Model III β (CI)**	**P-value**
**FBS (mg/dl)**									
	**Diet intervention (n = 48)**	-1.775 (-5.77, 2.22)	0.38	-1.84 (-5.89, 2.21)	0.369	-1.922 (-6.02, 2.17)	0.354	-1.952 (-6.092,2.18)	0.351
	**Supplement intervention (n = 49)**	-3.165 (-7.22, 0.897)	0.125	-3.149 (-7.23, 0.94)	0.13	-3.198 (-7.31, 0.92)	0.126	-3.16 (-7.332, 1.012)	0.136
**Insulin (μU/ml)**									
	**Diet intervention (n = 48)**	-17.02 (-47.08, 13.02)	0.263	-10.33 (-38.84, 18.17)	0.473	-6.127 (-34.19, 21.94)	0.666	-5.8 (-33.89, 22.29)	0.683
	**Supplement intervention (n = 49)**	-42.85 (-72.96, -12.73)	0.006	-47.52 (-75.87, -19.17)	0.001	-44.4 (-72.25, -16.64)	0.002	-45.3 (-73.23, -17.46)	0.002
**HOMA_IR**									
	**Diet intervention (n = 48)**	-4.043 (-9.721, 1.635)	0.161	-2.724 (-8.105, 2.675)	0.317	-1.985 (-7.29, 3.32)	0.456	1.944 (-7.26, 3.37)	0.470
	**Supplement intervention (n = 49)**	-8.5 (-14.235, -2.776)	0.004	-9.26 (-14.64, -3.873)	0.001	-8.67 (-13.97, -3.38)	0.002	-8.819 (-14.14, -3. 49)	0.001
**QUICKI index**									
	**Diet intervention (n = 48)**	-0.054 (-0.15, 0.047)	0.29	-0.063 (-0.165, 0.039)	0.222	-0.067 (-0.170, 0.036)	0.2	-0.073 (-0.176, 0.029)	0.159
	**Supplement intervention (n = 49)**	0.038 (-0.064, 0.14)	0.45	0.044 (-0.058, 0.147)	0.391	0.041 (-0.062, 0.145*)*	0.428	0.048 (-0.055, 0.152*)*	0.353
Model I: The p-value was calculated using ANCOVA and adjusted for BMI classification and energy difference. Model II: The p-value was calculated using ANCOVA, adjusted for BMI classification, energy difference, and age. Model III: p-value was calculated using ANCOVA adjusted for BMI classification, energy difference, age, and physical activity levels at the baseline. Supplement intervention: (curcumin and placebo [reference]), diet intervention: (DASH diet and standard diet [reference]). BMI: Body mass index, FBS: Fasting blood sugar, HOMA_IR: Homeostasis model assessment of insulin resistance, QUICKI: Quantitative insulin sensitivity check index, CI*: *Confidence intervals									

## 4. Discussion

The results of this study showed that the 12 wk DASH diet had no significant effect on glycemic parameters. Still, the simultaneous use of curcumin (1000 mg/day) can significantly reduce insulin levels (β = -45.3, 95% CI [-73.23, -17.46], p = 0.002) and IR (β = -8.819, 95% CI [-14.14, -3.49], p = 0.001). However, curcumin administration had no effect on FBS levels and QUICKI index.

IR and compensatory hyperinsulinemia play a major role in causing reproductive and metabolic disorders (3). Therefore, treatment by inducing insulin sensitivity and reducing IR effectively manages PCOS. According to meta-analysis results, curcumin supplementation may have beneficial effects on glycemic parameters (18). Previously, no randomized clinical trial has investigated the combined effect of the DASH diet and curcumin supplementation on glycemic parameters in normal-weight and overweight/obese women with PCOS who are candidates for IVF treatment. PCOS and obesity may have a synergistic detrimental effect on IR (19). However, not all PCOS patients with hyperinsulinemia are overweight or obese. The effects of diet and curcumin independent of weight status has not been investigated so far. In this study, mean insulin levels in normal weight groups were reduced to normal levels with curcumin intake. After adding curcumin to the intervention, the mean reduction of HOMA-IR was also higher in normal BMI groups. The amount of insulin clearance is low in obese people compared to lean people (20). The mechanisms enhancing the effect of curcumin can be explained by the insulin clearance hypothesis (21). Although decreased insulin clearance occurs to compensate and adapt to IR in the body, this condition is associated with dysglycemia. It may contribute to IR and diabetes through hyperinsulinemia-induced desensitization (5). Curcumin, with its antioxidant properties and its effect on increasing circulating adiponectin as an anti-inflammatory cytokine, can play an essential role in reducing the metabolic complications of PCOS (22). In patients with high adiponectin status, hepatic clearance of insulin is increased (23). Low circulating adiponectin levels were associated with IR, BMI, adiposity, and metabolic syndrome (22). In women with PCOS, the level of adiponectin is lower, while this change occurs regardless of adiposity (24). Another possible mechanism is related to the low-grade inflammation caused by PCOS and the antioxidant protective effects of curcumin (14).

Given that obesity and unbalanced dietary composition cause more disruption in insulin metabolism and inflammation (25), we therefore adjusted for BMI and energy difference and other relevant factors such as age (26) and physical activity (27). Based on the results of umbrella reviews of meta-analyses of RCTs, there was no high-conclusive evidence of an effect of diets alone on the metabolic and endocrine outcomes of PCOS. However, curcumin supplementation showed favorable effects on HOMA-IR (28). In the present study, after controlling for BMI, energy difference, age, and physical activity levels at baseline, the effect of curcumin on insulin levels and HOMA-IR remained significant. This indicates that the effect of curcumin on serum insulin and IR is independent of the effect of these factors.

In the present study, the same macronutrient components were considered for both diets. Regarding the macronutrient composition of the participant's diet at baseline, the mean protein intake in all groups ranged from 10.58–12.7, whereas we considered 18% protein in both diets. There is no optimal strategy for macronutrient distribution or dietary interventions in nutritional recommendations for PCOS patients (29). Recent evidence suggests that high protein intake can improve IR (30).

However, protein intake should not exceed 20% of total calorie intake (29). From the perspective of dietary interventions in our study, the results of Dixon's DASH diet index showed good adherence to the DASH diet. A meta-analysis of RCTs showed that the DASH diet can improve some glycemic parameters when implemented as a long-term intervention ( > 16 wk) (31). Therefore, the addition of curcumin may enhance the anti-inflammatory effects of the DASH diet leading to faster recovery of hyperinsulinemia, and improve IR.

All participants in both diets received personalized nutrition with identical macronutrient components and sodium prescription, while being offered several carefully analyzed menu items. Fortunately, participants showed good adherence to diet and curcumin intake. On the other hand, several adjustment models intend to confound factors of glycemic parameters to prevent misleading conclusions in data analysis and interpretation. On the other hand, several adjustment models for confounding factors of glycemic parameters have been considered to avoid misleading conclusions in data analysis and interpretation.

### Limitation

Dietary patterns can have long-term effects that could not be assessed due to the short duration of this study, which could lead to bias. Another source of bias could be related to seasonal food availability and preparation.

## 5. Conclusion

In conclusion, the present randomized clinical trials indicated that the co-administration of (1000 mg/day) curcumin and DASH diet for 12 wk, can have positive effects on reducing insulin levels, improving IR, and leading to faster recovery of hyperinsulinemia. Overall, the information presented here may highlight the potential of curcumin as a bioactive compound and future therapeutic target alongside a healthy diet for endocrine and metabolic diseases.

##  Data Availability

The data and materials of the current study are available from the corresponding author on reasonable request.

##  Author Contributions

H. Mozaffari-Khosravi, A. Aflatoonian, and T. Zohrabi designed and conducted the trial. A. Nadjaradeh and MH. Sheikhha provided scientific and technical support. H. Mozaffari-Khosravi and A. Aflatoonian supervised the study. S. Jambarsang carried out statistical analyses. T. Zohrabi interpreted the findings and drafted the manuscript. H. Mozaffari-Khosravi critically revised the manuscript. All authors read and approved the final version of the manuscript.

##  Conflict of Interest 

The authors declare that there is no conflict of interest.
